# Flexible Microwave Biosensor for Skin Abnormality Detection Based on Spoof Surface Plasmon Polaritons

**DOI:** 10.3390/mi12121550

**Published:** 2021-12-12

**Authors:** Junkai Bai, Hongfu Guo, Hua Li, Chen Zhou, Hanchao Tang

**Affiliations:** School of Physics and Optoelectronic Engineering, Xidian University, Xi’an 710071, China; bjk490385891@163.com (J.B.); xdu_lihua@163.com (H.L.); zhouchen0402@163.com (C.Z.); hctang1996@163.com (H.T.)

**Keywords:** flexible sensor, microwave biosensor, point of care test, health monitor, miniaturized device, spoof surface plasmon polaritons

## Abstract

Point-of-care testing plays an important role in the detection of skin abnormalities. The detection of skin abnormalities requires sufficient depth and no harm. A flexible microwave biosensor based on spoof surface plasmon polaritons was designed to meet the requirements of skin abnormalities. The designed biosensor, which works at 11.3 GHz, is small and can be flexibly attached to the skin surface of any part of the human body for measurement. The health status of the skin can be evaluated by the resonant frequency and the magnitude of the reflection coefficient of the sensor. The sensor was tested on pork skin. The experiment results showed that the sensor can detect skin abnormalities such as skin burn, skin tumor, and others. Compared with other sensors, the sensor has sufficient penetration depth because of the strong penetration of microwave electromagnetic waves. It is the first flexible microwave biosensor used for skin, which involves point-of-care testing, and continuous monitoring of skin.

## 1. Introduction

For a long time, skin health has been an important issue studied by researchers in the medical field. Common skin abnormalities, such as skin inflammation, skin burn, and skin cancer, pose a great threat to human health [1]. Timely and accurate examination of skin abnormalities in the early stages can often reduce the cost and difficulty of follow-up treatment. Therefore, the development of detection technology for skin abnormalities has long been an important research area. Traditional medical detection is often based on biopsy. Its detection time is lengthy and often requires complex instruments, which creates difficulties in meeting the demand for real-time and rapid detection. In addition, damage can be caused to the human body during biological tissue sampling. In light of this, development of point-of-care test (PoCT) biosensors has become a research hotspot in recent years. Doctors and patients can quickly learn about the situation of the disease through sensors. In recent years, researchers have designed many PoCT biosensors for a range of purposes, which has greatly improved detection efficiency in various medical fields such as blood glucose detection and nucleic acid detection [2,3,4,5]. However, there are relatively few PoCT biosensors for the detection of skin abnormalities. The only existing sensors for skin abnormality detection have many disadvantages, such as large volume, high cost, and others. Thus, researchers urgently need to design a flexible biosensor for skin abnormality detection.

In addition to the biochemical analysis of diseased tissues, traditional skin abnormality detection methods mostly rely on visual inspection. Visual observation is often subjective and the detection accuracy is relatively low. Infrared spectroscopy and confocal Raman spectroscopy can be used to detect skin abnormalities, but their wide application is limited by the high cost and long diagnosis time. At the same time, the penetration depth of light is low, so it is difficult to detect the area of the epidermis and dermis by infrared spectroscopy [6,7]. Ultrasonography can also be used to detect skin abnormalities, but its measuring probe is non-flexible and large, and the detection effect is poor when measuring the non-planar parts of skin [8]. The water content of skin reflects the health of the skin. Abnormal skin conditions such as skin inflammation, burns and skin cancer can be expressed by changes in skin water content [9]. Therefore, the detection of skin water content provides a new basis for the detection of skin abnormalities. Many skin biosensors detect skin abnormalities indirectly by measuring the water content of the skin. The flexible microwave biosensor we describe in this paper is based on the measurement of skin water content. A capacitance sensor is the commonly used sensor for measuring skin moisture content. A capacitive sensor for skin moisture detection is proposed in [10]. However, as for infrared spectroscopy, this method can only detect the stratum corneum of the skin. Therefore, the capacitive sensor is not suitable for the detection of skin abnormalities. In contrast to traditional detection methods, microwave electromagnetic waves can penetrate the epidermis and dermis of the skin for detection because of their low working frequency. Miniaturized near-field microwave probes have been widely used in the detection of human skin in recent years [11,12,13]. For example, the micromachined probe proposed in [14] works in the millimeter-wave band. The size of the probe is only 0.6 × 0.5 mm^2^. The sensor was fabricated by MEMS technology and has high measurement resolution. However, the biggest disadvantage of the near field microwave probe is that it is a single port structure and non-flexible circuit. When measuring, the probe needs to be fixed by hand, which is very inconvenient to operate. For some non-planar parts of skin, the non-flexible sensor cannot be effectively attached to the surface for measurement. Skin health monitoring is also a current focus in the medical field. In the process of skin recovery, wound healing can be evaluated in real time through continuous monitoring of the skin. The non-flexible circuit structure, however, makes it impossible to continuously adhere to the skin surface to monitor the skin state. Many flexible skin biosensors have been developed in recent years. These sensors cover many fields, such as temperature sensors, pressure sensors, vital signs sensors, and others [15,16,17,18,19]. However, flexible biosensors for skin abnormalities based on microwave technology have not been proposed. Unlike traditional flexible sensors, flexible microwave biosensors have a deep penetration depth into the skin. By designing a microwave sensor with a specific size, the desired depth of skin can be detected. Moreover, microwave sensors are non-destructive and will not cause harm to the human body in the detection process. The processing technology of such sensors is also simple, and the production costs are low, which makes them suitable for large-scale production.

The flexible microwave sensor designed in this paper is based on spoof surface plasmon polaritons (SSPPs). SSPPs evolved from surface plasmon polaritons (SPPs) which are surface waves (SWs) that exists at the interface between two materials with different properties. SPPs are excited at an interface between metal and dielectric. Metal has negative permittivity and dielectric has positive permittivity which enables them support SPPs. SPPs are excited by the coupling of the electromagnetic field and the collective oscillations of surface electrons on the surface of the metal [20]. The amplitude of the electromagnetic field decays exponentially as the distance from the interface increases. Since the electromagnetic field is concentrated at the interface, SPPs are more sensitive to surface conditions, which makes them more suitable for sensor design. Therefore, many sensors based on SPPs have been designed over many years [21,22,23,24,25,26,27,28,29]. Satyendra Kumar Mishra et al. [25] designed an SPR-based sensor for aqueous samples with high sensitivity. Pavel Adam et al. [27] and K Matsubara et al. [28] proposed a surface plasmon sensor for detecting the thickness of the bovine albumin layer and the concentration of ethanol in alcohol, respectively. Dong Cheng et al. [29] designed an SPPs biosensor working in the terahertz band, which can be used to detect viruses. The sensor has high sensitivity, and it can detect avian influenza viruses (e.g., H5N2, H1N1, H9N2). Spoof surface plasmon polaritons (SSPPs) were invented in 2004, and work in the microwave band with similar properties to surface plasmon methods [30]. In recent years, many microwave devices based on SSPPs have been proposed, such as filters and antennas [31,32,33,34,35]. SSPPs can be designed as various types of filter because of their stopband characteristics [36,37,38,39]. Yiqun Liu et al. [36] proposed a bandpass filter with controllable bandwidth based on high-order SSPPs. Dong-Fang Guan et al. [39] proposed a hybrid SIW-SPP bandpass filter. Furthermore, SSPPs can be used to excite resonance modes of resonance unit because of their unique SW mode and structure, which means that they can be designed for many new types of microwave device [40,41,42,43]. Xiaoqing Yang et al. [40] designed an SSPPs-based sensor for detecting defects of metal. Hongxin Zhao et al. [43] designed a tri-band band-pass filter based on SSPPs. In terms of the sensor design, the sensitivity of an SSPPs-based sensor can be improved because the electromagnetic field is mainly concentrated on the surface of metal. Furthermore, the influence on the measurement results will be reduced when other non-measured objects approach or touch the sensor. Sensor arrays are measuring devices composed of many sensor units and can detect many positions at the same time. The low crosstalk characteristics of SSPPs plays an important role in designing sensor arrays, which increases the density of the sensor unit and measurement efficiency. In addition. SSPPs can propagate along flexible and ultra-thin dielectric media and, therefore, can be designed as flexible printed circuits (FPCs) [44]. The many advantages of SSPPs make them very suitable for the design of skin sensors. However, there have been few skin sensors based on SSPPs to date. Based on the structure of SSPPs, a flexible microwave biosensor working at 11.3 GHz was designed and is reported in this paper. According to the author’s knowledge, the sensor is the first flexible microwave sensor for human skin abnormality detection and monitoring. The sensor can be effectively attached to the human body surface to detect the skin and can also monitor the skin for a long time. Unlike other sensors, the sensor can detect the skin using microwave technology, and can detect the epidermis and parts of the dermis without damage. In addition, the resolution of the sensor is high because of its small size and the processing technology of the sensor is simple. The sensor has great advantages in PoCT and the monitoring of skin diseases.

## 2. Theory and Design of the Sensor

### 2.1. Structure and Principle of Sensor

**Figure 1:**
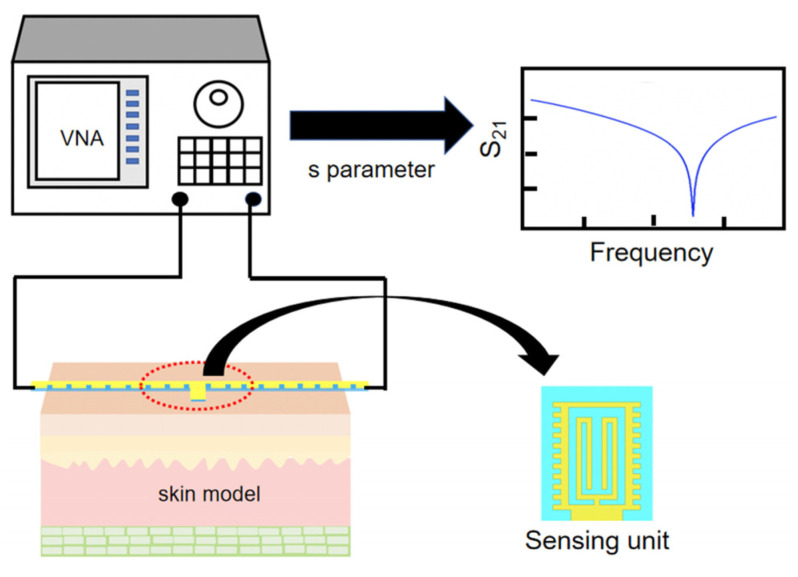
Working principle of sensor.

Figure 1 shows the working principle of the sensor. Material under test (MUT) contacts the sensor during measurement, and its two ends are connected to the vector network analyzer (VNA) through a coaxial line. The dielectric properties of MUT can be obtained by analyzing the changes of S parameters.

**Figure 2:**
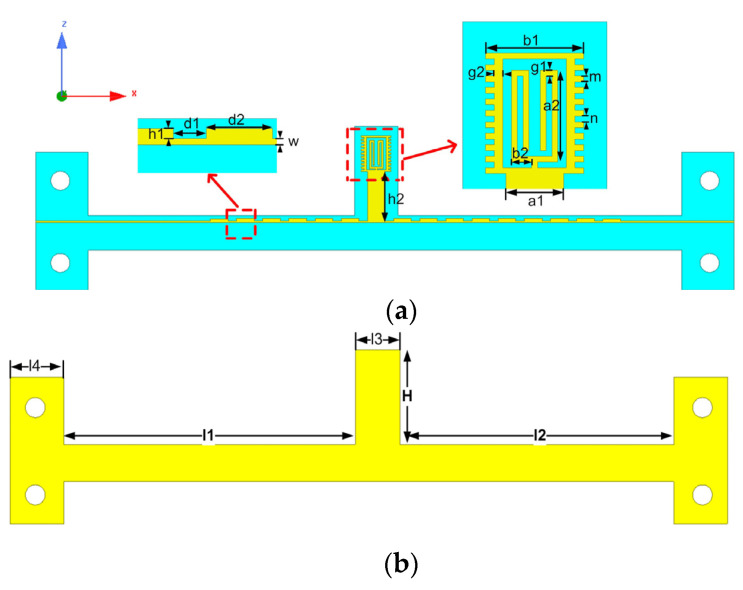
(**a**) Top view of sensor; (**b**) Bottom view of sensor.

The sensor described in this paper is based on SSPPs, which is a kind of electromagnetic surface wave propagating along the metal interface caused by the interaction between free electrons and photons. Figure 2a,b shows the structure of the upper and lower surfaces of the sensor based on SSPPs. To achieve the flexible structure of the transmission line, the sensor is designed on a 0.1 mm thick polyimide substrate (relative permittivity of 3.447 and loss tangent of 0.0027) with a 0.018 mm thick copper layer. The whole transmission line is 80 mm long, 4 mm wide and is excited by the microstrip line. The above parameters in Figure 2a,b are set as H = 9.5 mm, l1 = 30.5 mm, l2 = 32.5 mm, l3 = 5 mm, l4 = 6 mm, h1 = 0.3 mm, h2 = 5.7 mm, d1 = 1 mm, d2 = 2 mm, w = 0.2 mm, a1 = 2 mm, a2 = 3.2 mm, b1 = 3.4 mm, b2 = 0.7 mm, g1 = 0.2 mm, g2 = 0.3 mm, m = 0.2 mm, *n* = 0.2 mm. Cylindrical holes at both ends of the transmission line are used to secure the SMA connector. The SSPPs circuit is mainly composed of a rectangular unit and a sensing unit located at its center. The size of the sensing unit is only 4.3 × 3.4 mm^2^ and the transmission line is only 0.2 mm wide, so that the sensor has high resolution. The transmission zero point of the microwave sensor is also generated by the sensing unit, which acts as the main sensing area and traps the main electromagnetic energy in this area.

**Figure 3:**
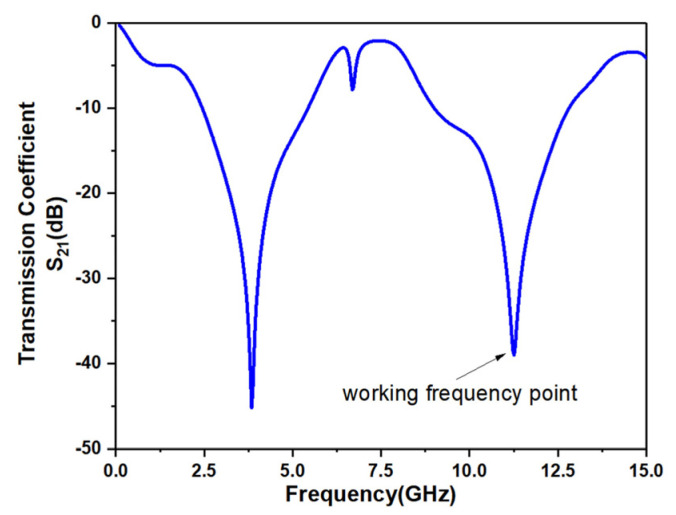
Simulation result of sensor without MUT loaded.

The S parameters of the sensor are simulated by ANSYS Electronics Desktop software. Figure 3 shows the S parameters of the sensor when MUT is not loaded. It can be seen from the figure that the sensor has two resonant frequency points in the range of 2 GHz–14 GHz, which are generated by the sensing unit. When the dielectric properties of MUT change, these resonant frequency points will change accordingly. The change of MUT dielectric properties can be analyzed by observing the change of resonant frequency point. The second resonant mode is used as the measurement frequency point, which works at 11.3 GHz. Compared with the first resonant mode, which works at 3.9 GHz, the second resonant mode has better measurement sensitivity and a wider measurement range.

**Figure 4:**
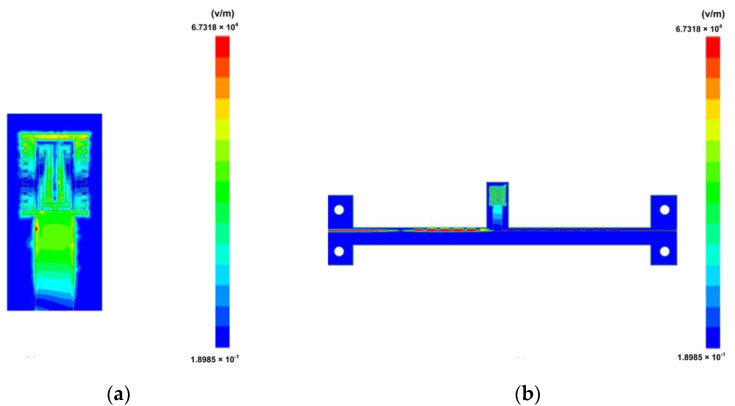
(**a**) Electric field of sensing unit; (**b**) Electric field of transmission line.

Figure 4a,b show the electric field energy distribution of the sensor working at 11.3 GHz. It can be seen from the figure that when the sensor works at the resonant frequency, the electric field energy is concentrated at the sensing unit. The electromagnetic wave propagating in the x-direction is confined to the sensing unit. The resonant effect of the sensing unit on the electromagnetic wave makes the transmission line form a transmission zero point. MUT with different dielectric properties will have different effects on S parameters of the sensor. The working principle of the sensor can be explained by perturbation theory [45]:

(1) $$\frac{\Delta f_{r}}{f_{r}}=\frac{\int_{v_{c}}\left(\Delta \varepsilon E_{1}\cdot E_{0}+\Delta \mu H_{1}\cdot H_{0}\right)dv}{\int_{v_{c}}\left(\varepsilon_{0}{\left|E_{0}\right|}^{2}+\mu_{0}{\left|H_{0}\right|}^{2}\right)dv}$$

where $f_{r}$ and $\Delta f_{r}$ are resonant frequency and the change of resonant frequency, respectively, $v_{c}$ is the volume of MUT, $\varepsilon_{0}$ and $\mu_{0}$ are dielectric property without MUT loaded, Δε and Δμ are change of dielectric property when MUT is loaded on the sensor to perturb volume $v_{c}$, $E_{0}$ and $H_{0}$ are the electric field and magnetic field without MUT loaded, while $E_{1}$ and $E_{2}$ are the electric field and magnetic field when MUT is loaded on the sensor to perturb volume $v_{c}$. Furthermore, Equation (1) can be simplified to Equation (2) because Δμ of skin is zero.

(2) $$\frac{\Delta f_{r}}{f_{r}}=\frac{\int_{v_{c}}\left(\Delta \varepsilon E_{1}\cdot E_{0}\right)dv}{2\int_{v_{c}}\left(\varepsilon_{0}{\left|E_{0}\right|}^{2}\right)dv}$$

The sensing unit designed in this paper mainly evolved from the short rectangular unit. The dispersion curve of SSPPs with the rectangular unit can be determined by the Equation (3) [46]:

(3) $$\sqrt{\beta^{2}-k_{0}{}^{2}}=k_{0}\left(\frac{a}{p}\right)\tan\left(k_{0}h\right)$$

**Figure 5:**
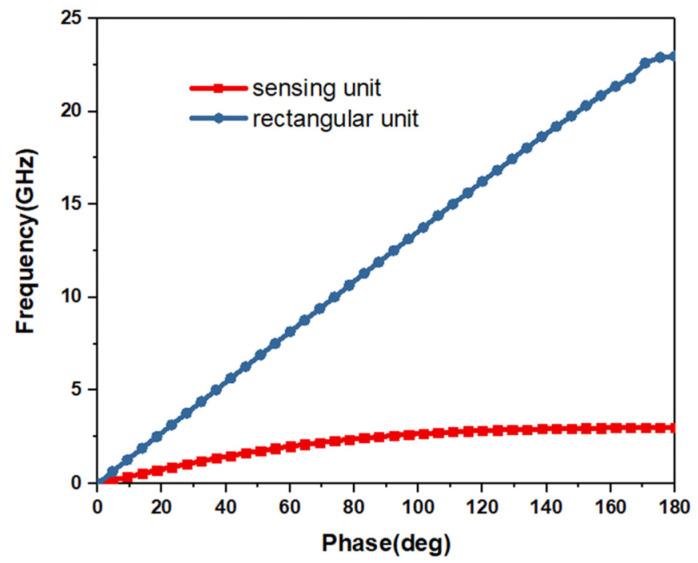
Dispersion plots of sensing unit and rectangular unit.

**Figure 6:**
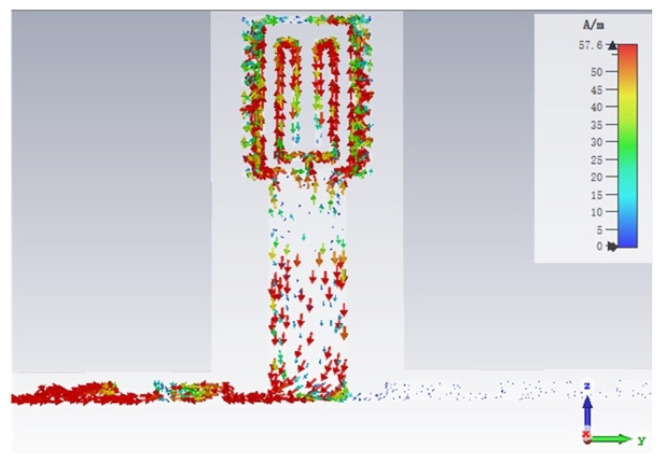
The current distribution of sensing unit.

The *k*_0_ and *β* are vacuum wave number in free space and SPPs wave number. And *h*, a, and p represent depth, width and length of the rectangular unit, respectively. The groove depth of the rectangular unit determines the asymptotic frequency of SSPPs. The deeper the depth, the lower the asymptotic frequency. The sensing unit evolved from the rectangular unit located at the middle of the transmission line. Figure 5 shows the dispersion of the rectangular unit and sensing unit, respectively. It can be seen from the figure that the asymptotic frequency of the rectangular unit is 23 GHz, while the asymptotic frequency of the sensing unit is 3 GHz. The length of the current path was effectively increased by greatly lengthening the groove depth of the rectangular unit and the inductance of the unit is also increased. The current path is further extended by etching the rectangular defected pattern at the end of the rectangular unit which further increases the inductance. Increasing the gap between conductors of the sensing unit can produce a greater capacitance effect. To increase the capacitance of the sensing unit, a small rectangular gap on both sides of the rectangular unit is etched. A U-shaped metal strip is designed at the rectangular defected pattern to improve the sensitivity of the sensor. Due to the increase in capacitance and inductance, the resonant frequency of the sensor decreases, which also makes the size of sensing unit smaller. Figure 6 shows the current distribution of the designed sensing unit at 11.3 GHz. It can be seen that the current accumulates from the transmission line to the sensing unit.

**Figure 7:**
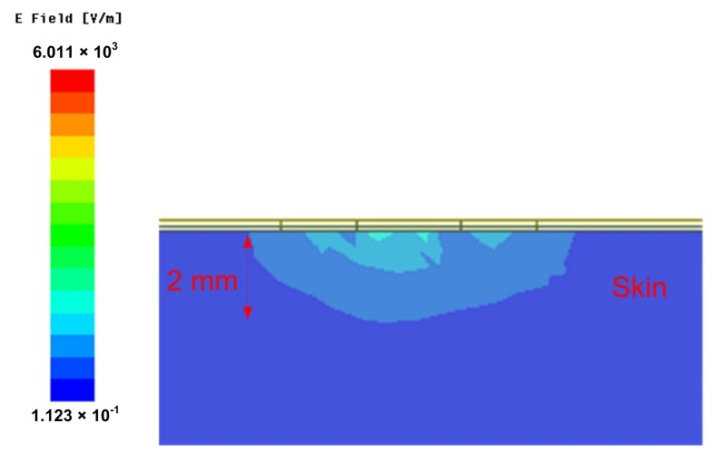
The penetration depth of sensor.

The influence of subcutaneous tissue, such as fat and muscle, on detection results should be avoided as far as possible when the sensor detects the skin. Therefore, the penetration depth of the sensor should be controlled within the thickness of skin. Figure 7 shows the simulated penetration depth of the sensor designed working at 8.8 GHz. For the skin model in Figure 7, the relative permittivity is 30 and the loss tangent is 0.3. Figure 7 shows the electromagnetic wave of the sensing unit coupled to the skin. The electric field reduces to 8.016 × 10^2^ V/m level at 2 mm depth, which means the effective detection depth is less than 2 mm.

### 2.2. Equivalent Circuit Model of Sensor

**Figure 8:**
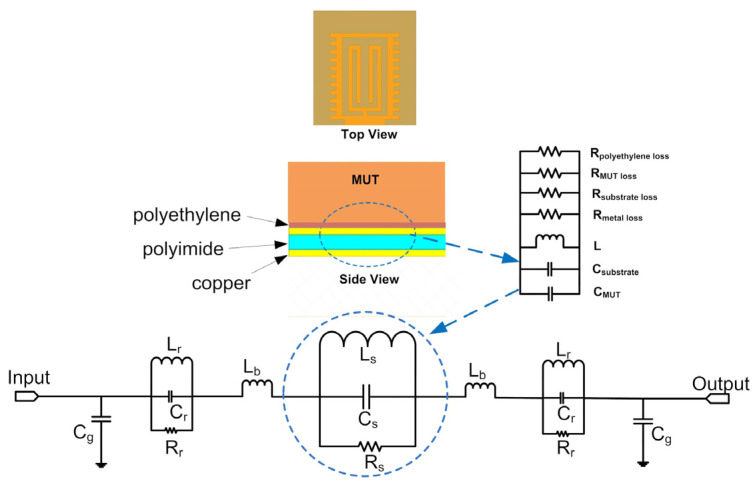
Equivalent circuit model of sensor.

Figure 8 shows the equivalent circuit model of the sensor. The mechanism of the sensor can be explained by the equivalent circuit. In Figure 8, L_r_, C_r_ and R_r_ represent the equivalent inductance, capacitance and resistance of the rectangular unit, respectively. The width and groove depth of the rectangular unit affect its inductance and capacitance. The parallel resonant circuit, composed of L_r_, C_r_ and R_r_, determines the high-frequency cut-off characteristics of the rectangular unit. L_b_ represents the equivalent inductance of the feeding microstrip line and the metal strip between adjacent rectangular units, which has little effect on the resonant frequency of the sensor. C_g_ represents the equivalent capacitance between the metal conductor on the upper surface and the floor. The main sensing area of the whole sensor circuit is the sensing unit in the middle. As shown in Figure 8, the equivalent circuit of the sensing area can be composed of a parallel resonant circuit which comprises L_s_, C_s_ and R_s_. L_s_, C_s_ and R_s_ correspond to the equivalent inductance, equivalent capacitance and equivalent resistance of the sensing unit, respectively. The equivalent capacitance C_s_ is composed of the equivalent capacitance C_substrate_ and the equivalent capacitance C_MUT_, which represent the capacitance between conductor and medium and the capacitance between conductor and MUT, respectively. The resonant frequency in this equivalent model can be expressed as Equations (4) and (5):

(4) $$f=\frac{1}{2\pi \sqrt{L_{s}C_{s}}}$$

(5) $$C_{s}=C_{substrate}+C_{MUT}$$

When the dielectric properties of MUT change, the C_mut_ also changes, which results in a shift of resonant frequency. The resonant frequency of the sensor is mainly determined by L_s_ and C_s_, and the influence of other lumped elements is small. A change of dielectric properties of MUT can be obtained by analyzing the change of resonant frequency. The dielectric properties of MUT also affect the magnitude of the reflection coefficient as well as the shift of resonance frequency. The loss tangent is the main factor that affects the magnitude of the reflection coefficient. The higher the loss tangent, the higher the magnitude of the reflection coefficient. Therefore, the magnitude of the reflection coefficient can also be used to reflect the change of the dielectric properties of MUT.

Different MUT have different dielectric properties. For biological tissues such as skin, fat and muscle, the difference of water content will lead to different permittivity and loss tangents. The higher the water content, the higher the permittivity. In addition, the loss tangent usually increases with increase in water content. Under the influence of permittivity and loss tangent, the skin with higher water content often has lower resonant frequency and a higher magnitude of the reflection coefficient. Compared with the sensor without MUT loading, the resonant frequency of the sensor loaded with high water content MUT tends to have low frequency and a high magnitude of the reflection coefficient. Generally, the water content of skin tumors and skin with inflammation is higher, while the water content of burned skin is lower. When different MUT are loaded on the sensing unit of the sensor, the resonant frequency and magnitude of reflection coefficient will change accordingly. Usually, there is a significant difference in the water content of skin between healthy skin and unhealthy skin, and the measured resonant frequencies and magnitude of reflection coefficient are quite different. Using this difference, healthy skin and unhealthy skin can be distinguished. The characteristic of resonant frequency and magnitude of reflection coefficient changing with dielectric property means that the sensor can be used to detect and monitor skin abnormalities. The sensor is placed on different parts of skin for measurement, and the unhealthy parts of the skin can be identified by different resonant frequencies and magnitude of reflection coefficients obtained from the measurement. Furthermore, the skin can be monitored by the sensor in real time.

### 2.3. Measurement Operation

**Figure 9:**
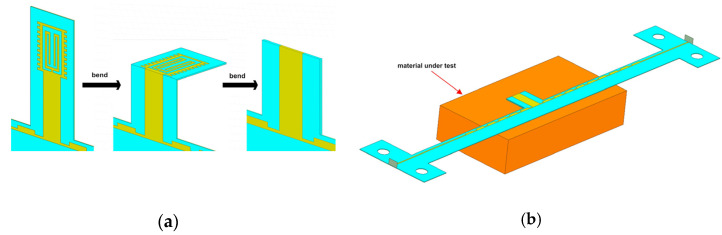
(**a**) Operation before measurement; (**b**) Measurement operation diagram.

To ensure the accuracy of measurement, MUT should only contact the sensing unit of the sensor and not part of the transmission line. As shown in Figure 9a, the sensing unit is bent to the back of the sensor for measurement to prevent MUT from contacting the transmission line. In addition, a 0.01 mm PE film is placed on the surface of MUT to avoid direct contact between MUT and the sensor. To improve the stability of operation during measurement, the sensing unit can be fixed to the back floor with glue. Figure 9b shows the measurement process of the sensor when MUT is placed on the sensing unit. The resonant frequency decreases and the magnitude of reflection coefficient increases with the increase in permittivity and loss tangent. Unlike the complex detection methods of traditional skin detection, the skin detection of the sensor can be achieved directly through the S parameters. The simple measurement method of the sensor makes it possible to realize PoCT. The sensor has great potential for improving detection efficiency and speed.

## 3. Measurement and Discussion

**Figure 10:**
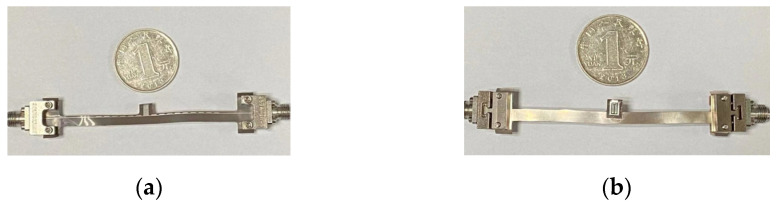
(**a**) Top view of sensor; (**b**) Bottom view of sensor.

**Figure 11:**
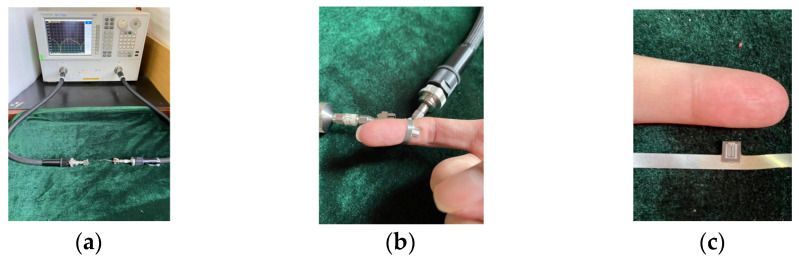
(**a**) Measurement operation of sensor; (**b**) Wrap the finger; (**c**) Sensing unit of the sensor.

The designed microwave sensor is shown in Figure 10a,b. The size of the sensing unit is only 0.6 × 0.5 mm^2^, which allows for the higher resolution of the sensor. The substrate of the sensor is 0.1 mm thick polyimide. As shown in Figure 11b,c, the sensor has good flexibility and can be effectively attached to the skin for measurement. The sensor can readily measure any part of the human body because of its flexibility, which has great advantages for improving the detection convenience of the sensor. Pork skin was used as experimental material to replace human skin in this research, which has similar dielectric properties to human skin. All the pork skin used in this article was obtained from a pork food company located at Zhangba East Road, Yanta District, Xi’an, Shanxi, China.

**Figure 12:**
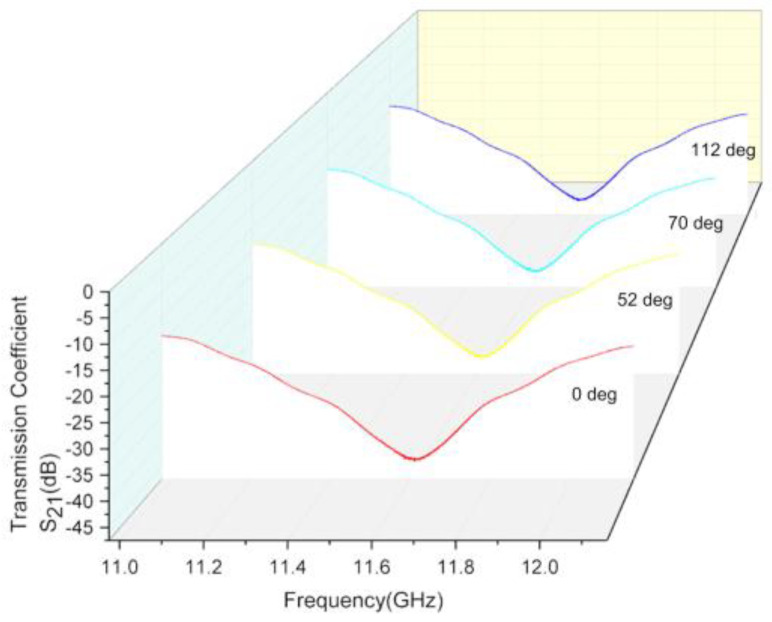
Measurement results when sensor bent to different curvatures.

The curvature of the sensor has little effect on its resonant frequency and magnitude of reflection coefficient. As shown in Figure 12, the resonant frequency of the sensor bent to 0°, 52°, 70° and 112°, is almost 11.6 GHz. The magnitude of the reflection coefficient of the sensor bent to the above four curvatures is almost −45 dB. The sensor can be bent to any curvature to measure the skin because of its insensitivity to curvature.

**Figure 13:**
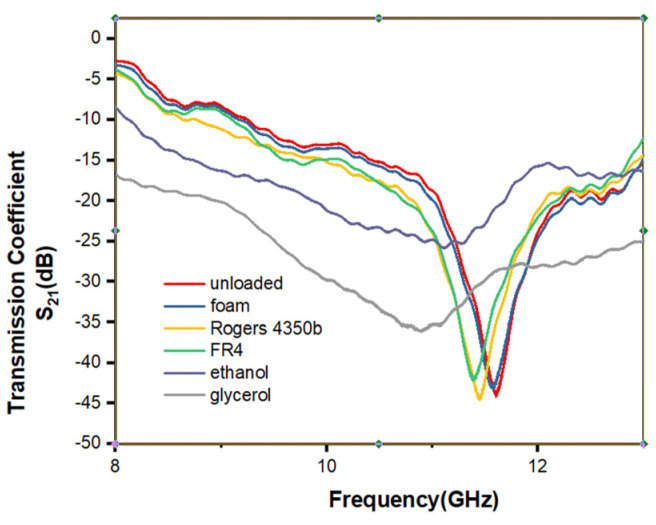
Measurement results when sensor is loaded with MUT with different dielectric properties.

Figure 13 shows the S parameters of the sensor when the sensor is loaded with MUT with different dielectric properties. As the permittivity increases, the resonant frequency moves towards low frequency.

**Figure 14:**
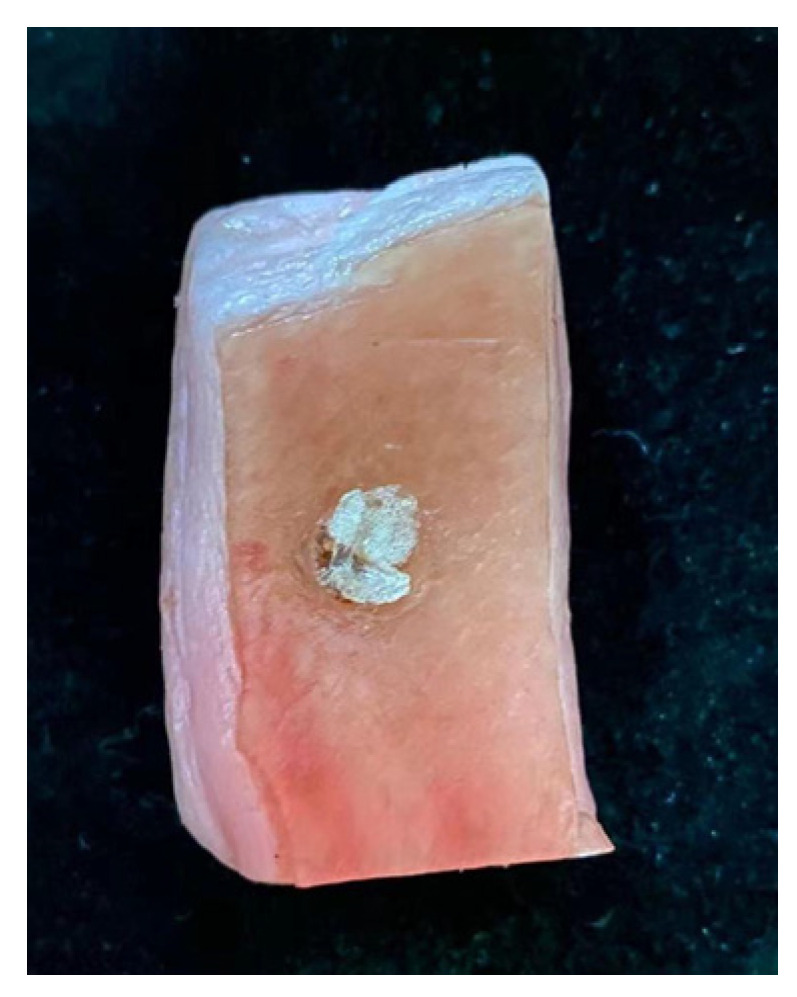
Pork skin with abnormal tissue mimicking phantom materials.

**Figure 15:**
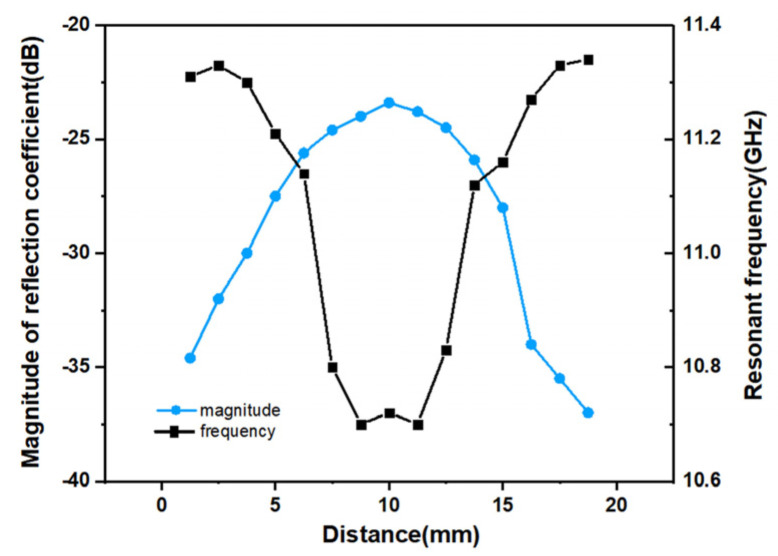
The measurement results of one-dimensional direction for abnormal tissue detection.

**Figure 16:**
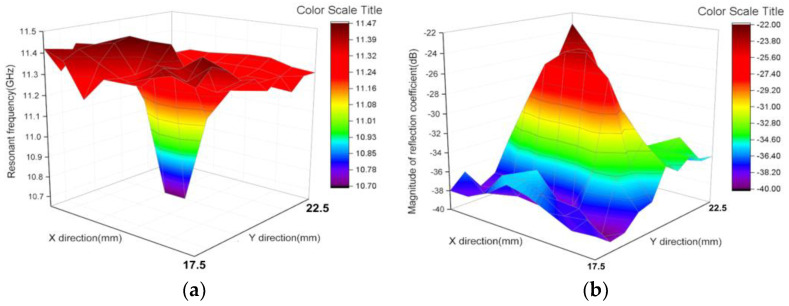
The measurement results of two-dimensional direction for abnormal tissue detection: (**a**) Images using resonant frequency; (**b**) Images using magnitude of reflection coefficient.

An experiment simulating the detection of abnormal skin tissue was performed. As shown in Figure 14, pork skin was used as the model for human skin because of its dielectric properties that are similar to human skin. Abnormal tissue mimicking phantom materials was made to mimic abnormal tissues with high water content (such as malignant skin, inflammatory skin, etc.) and it was embedded into the pork skin as abnormal skin tissues [47,48]. A lateral measurement which was carried out in a one-dimensional direction was performed and the result is shown in Figure 15. As shown in Figure 15, the motion resolution was 1.25 mm and the abnormal tissue can be clearly identified. Furthermore, another measurement which was carried out in a two-dimensional direction was performed. The motion resolution of the sensor was set to 2.5 × 2.5 mm^2^ for the convenience of measurement. As shown in Figure 16a,b, the position with dark color represents the position with high permittivity and high loss tangent, which means the water content was high. The deepest color in the middle of the images corresponds to the abnormal tissue of the skin, and therefore the abnormal tissue can be roughly distinguished. This experiment showed that the designed sensor can detect skin abnormalities.

**Figure 17:**
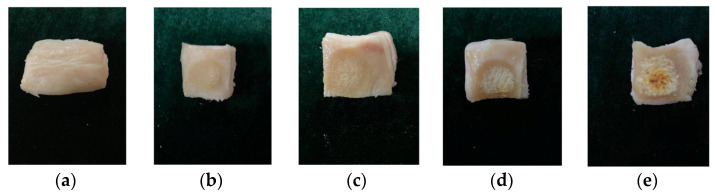
Burns of different degrees: (**a**) burned for 0 s; (**b**) burned for 40 s; (**c**) burned for 80 s; (**d**) burned for 120 s; (**e**) burned for 160 s.

**Figure 18:**
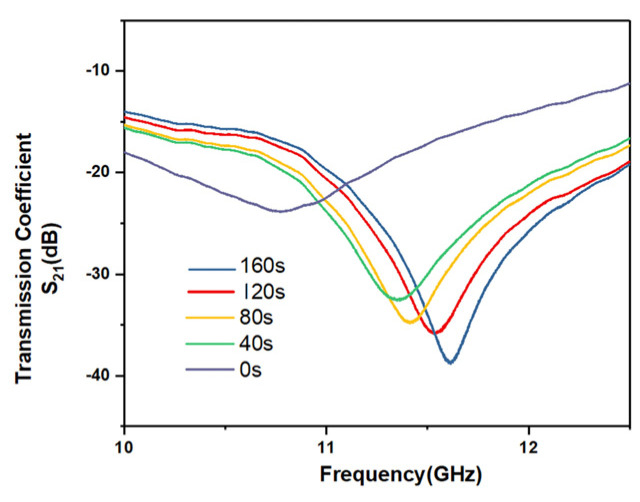
Measurement results of burns of different degrees.

Skin burn is a common skin injury. At present, the most common detection method for burn examination relies on visual inspection. The measurement results of the visual inspection are often subjective, and the measurement accuracy is very low, of the order of only 64–76% [49]. The sensor designed in this paper uses microwave technology to detect the wound effectively. In addition, the sensor can detect the epidermis and part of the dermis without damage because of the penetration effect of microwave electromagnetic waves. In general, burns reduce the water content of the skin. Different degrees of burn will reduce the water content of the skin to different degrees, and the resonant frequency will also be different. In order to verify the detection ability of the sensor for skin burns, five pork skin samples heated to different degrees for measurement were used to mimic skin burns to different degrees. The pork skin was heated by hot air gun and the four samples were heated for 40 s, 80 s, 120 s and 160 s respectively. The samples with different degrees of burns represent wounds with different degrees of injury. As shown in Figure 17, the differences in pork skin with different heating was very obvious. The unheated skin in Figure 17a had a smooth surface and the skin gradually burned with the deepening of heating. Figure 18 shows the measurement results: the four pork skin samples with different degrees of burns had different resonant frequencies and magnitude of reflection coefficients. The unburned skin had the lowest resonant frequency and the highest magnitude of reflection coefficient. The resonant frequency gradually increased and the magnitude of reflection coefficient decreased with the deepening of the burning. The degree of skin burn can be evaluated by the resonant frequency and the magnitude of the reflection coefficient.

**Figure 19:**
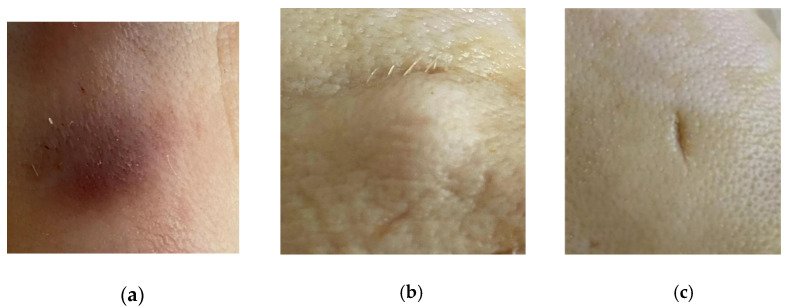
Different abnormal skin abnormalities: (**a**) bruise; (**b**) nodules; (**c**) wound.

**Figure 20:**
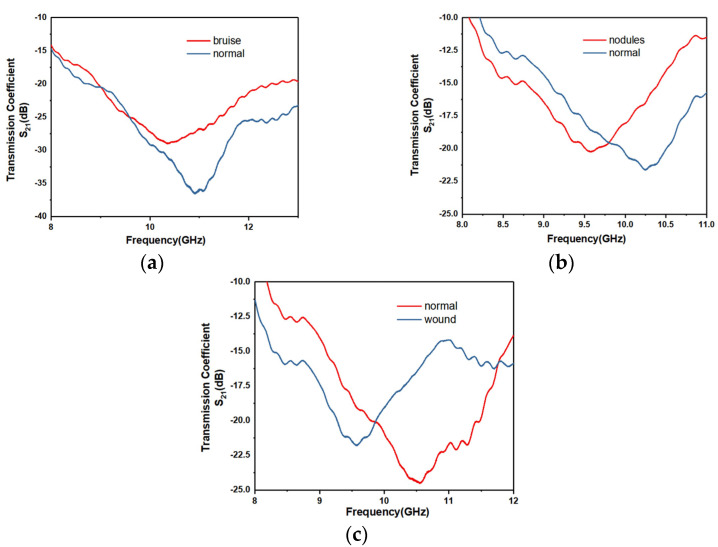
Measurement results with different abnormal skin abnormalities: (**a**) bruise; (**b**) nodules; (**c**) wound.

The sensor can also be used to diagnose common abnormal skin abnormalities, such as bruises, nodules and wounds. An experiment using pork skin was performed. The results of the following three experiments, which measured bruises, nodules and wounds, respectively are shown in Figure 19. As shown in Figure 20, the resonant frequency and the magnitude of reflection coefficient of the abnormal part of skin measured by the sensor were quite different from the normal part of the skin. The abnormal skin tissue usually had a higher magnitude of reflection coefficient and lower resonant frequency than normal skin. The experiments show that the sensor designed has the ability to diagnose skin diseases and that it has potential in PoCT.

## 4. Conclusions

A flexible microwave sensor for the detection of skin abnormalities based on spoof surface plasmon polaritons was designed in this research. The sensor can be effectively attached to the human body for measurement and the measurement is non-invasive. The size of the sensing unit is 0.6 × 0.5 mm^2^, which provides a small size and relatively high resolution of the sensor. Some experiments including abnormal tissue detection and skin burn detection were performed. Skin abnormalities can be detected and monitored by the resonant frequency and magnitude of the reflection coefficient of the sensor. The efficiency of measurement has been greatly improved because of the simple measurement method and the flexible circuit form of the sensor. It has great potential in PoCT of skin abnormalities.

## Data Availability

Not applicable.
